# The expression of *VDACs* and *Bcl2* family genes in pituitary adenomas: clinical correlations and postsurgical outcomes

**DOI:** 10.3389/fendo.2024.1481050

**Published:** 2024-10-10

**Authors:** AN Facundo, M Magalhães, GC Nascimento, RS Azulay, RM Santos, LA Freitas, AGPAC Nascimento, VP Rodrigues, WC Santos, AMGS Beckman, JMF Abreu, RP Silva, EL Carneiro, CP Oliveira Neto, RM Gil da Costa, R Corcoy, E Mato, MS Faria

**Affiliations:** ^1^ Post-Graduate Program in Adult Health (PPGSAD), Federal University of Maranhão (UFMA), São Luis, Brazil; ^2^ Service of Endocrinology, University Hospital of the Federal University of Maranhao (HUUFMA), São Luis, Brazil; ^3^ Research Group in Clinical and Molecular Endocrinology and Metabology (ENDOCLIM), Federal University of Maranhão (UFMA), São Luis, Brazil; ^4^ Service of Radiology, University Hospital of the Federal University of Maranhao (HUUFMA), São Luis, Brazil; ^5^ Service of Pathology, University Hospital of the Federal University of Maranhao (HUUFMA), São Luis, Brazil; ^6^ Department of Morphology, Federal University of Maranhao (UFMA), São Luis, Brazil; ^7^ Service of Neurosurgery, University Hospital of the Federal University of Maranhao (HUUFMA), São Luis, Brazil; ^8^ CIBER Bioengineering, Biomaterials and Nanotechnology (CIBER-BBN), Instituto de Salud III, Madrid, Spain; ^9^ Department of Nutricion and Endocrinology of Institut de Recerca de l'Hospital de la Santa Creu i Sant Pau, Barcelona, Spain

**Keywords:** pituitary adenoma, VDAC, bax, BAK, apoptosis, tumor regrowth

## Abstract

**Introduction:**

Pituitary adenomas (PAs) are benign tumors with high prevalence and, occasionally, aggressive course. The tumorigenesis of these lesions is not completely understood at the molecular level. BAK1 and BAX proteins play fundamental roles in apoptosis and seem to interact with VDAC proteins, whose expressions have been markedly altered in cancer, impacting their prognosis.

**Objective:**

to evaluate the gene expression of *VDAC1, VDAC2, BAK1* and *BAX* and their association with clinical and imaging characteristics in PA.

**Methods:**

Clinical-epidemiological data were collected from 117 tumor samples from patients affected by PA. Invasiveness was assessed by the Knosp scale. Gene expression was examined by real-time PCR. Relative expression analysis was performed by 2^(-DDCt) method.

**Results:**

The sample was mainly composed of women (69/117 – 57.2%). Tumor subtypes observed were Non-Functioning (NF) (73/117 – 62.4%), Acromegaly (24/117 – 20.5%) and Cushing’s Disease (CD) (20/117 – 17.1%). Compared to normal tissue, there was a significant reduction in *VDAC1* expression in the Acromegaly (p=0.029) and NF (p=0.002) groups. *BAX* expression was lower in all groups (p <0.001; p=0.007; P =0.005). No difference was found in *VDAC2* and *BAK1* expression, compared to normal pituitary. Overexpression of *VDAC2* occurred in PAs with post-surgical regrowth (p=0.042). A strongly negative correlation was observed in *BAX* and *BAK1* expression in CD.

**Conclusion:**

The results indicated that downregulations of *VDAC1* and *BAX* may be related to resistance to apoptosis. In contrast, overexpression of *VDAC2* in regrowing PAs suggests an antiapoptotic role for this gene. In summary, the genes evaluated might be involved in the biopathology of PAs.

## 1 Introduction

Pituitary adenomas (PAs) constitute a heterogeneous group of lesions with a mostly benign clinical course, and the global prevalence of PAs is estimated to be 89.1/100,000 (1). Although only 0.2% of these tumors have metastatic potential (2), a considerable percentage have aggressive characteristics, defined by local invasiveness, resistance to clinical-surgical treatment, and high growth potential (3–5).

The most recent classification proposed by the WHO establishes different subtypes of PAs according to histological and hormonal characteristics, in addition to identifying transcription factors, such as pituitary-specific transcription factor (PIT1), T-box transcription factor (TPIT) and steroidogenic factor 1 (SF1), some of which are associated with a more aggressive clinical course (6). However, the role of molecular markers classically used in other tumors, such as Ki-67 and p53, is not completely known in the (7, 8) and better results in the clinical management of PAs require a greater understanding of their biopathology and the identification of more efficient prognostic biomarkers, potentially including those involved in pathological processes that lead to the acquisition of cancer characteristics, such as resistance to cell death by apoptosis (9).

In this context, studying the role of key components of the cell death process may be fundamental to understanding the pathogenesis of PAs (10). Among them, the proteins BCL-2-associated X protein (BAX) and BCL-2 antagonist killer 1 (BAK1) are important regulators of the decisive step of mitochondrial outer membrane (MOM) permeabilization in apoptosis. The inhibition of these proteins has been reported in different cancers, such as breast, lung and hematological neoplasms (11, 12).

More recently, an important role for voltage-dependent anion channel proteins (VDACs) in the regulation of cell death has been suggested (13, 14). VDACs represent almost 10% of the MOM and appear to interact with BCL-2 family proteins to control the cell death process (15). To date, three VDAC isoforms have been described in mammals. VDAC1 was identified in a greater number of tissues, has a greater capacity for the molecular transport of electrolytes and ATP, and appears to play a role in inducing cell death in these tissues (16).

In some malignant tumors, *VDAC1* overexpression has been found to be associated with important element of the clinical course (17, 18). Jóźwiak et al. reported greater *VDAC1* expression in endometrial tumor cells than in normal tissue (19). In tumors of the esophagus, colon and prostate, reduced *VDAC1* expression was associated with decreased cell proliferation and an improved therapeutic response (20–22).

VDAC2 acts by recruiting BAK and BAX proteins to the MOM and inhibiting BAK-mediated apoptosis by forming complexes with its inactive form, suggesting an antiapoptotic role for this protein (23). Studies have demonstrated that interaction with VDAC2 is essential for the induction of BAX-mediated apoptosis but may be dispensable for BAK1-mediated apoptosis (24). In thyroid tumors, Mato et al. showed an up-regulation of *VDAC2* expression in all histological subtypes of tumors analyzed. However; *BAX* and *BAK1* genes showed a strong down-regulation and its silencing appears to lead to a better response to chemotherapy (25).

Although studies on this subject have indicated a possible impact of changes in the expression of *VDAC1* and *VDAC2* on the biological behavior of some malignancies, definite results are scarce and still preliminary (19, 21, 26, 27). To our knowledge, data on the expression of *VDACs* in benign tumors, particularly PAs, are lacking. Moreover, the inhibition of apoptosis has been associated with aggressiveness in other tumors and may be involved in the aggressiveness of PAs (28).

In this scenario, the role of VDACs and their possible interaction with BCL-2 family proteins in the development and progression of these tumors has not been studied to date, and the identification of reliable clinical markers for PAs remains a challenge. The aim of this study was to evaluate the gene expression of *VDAC1, VDAC2, BAX* and *BAK1* in a group of patients with diagnosis with different subtypes of PAs and correlate their expression levels with the clinical characteristics of aggressiveness and invasiveness.

## 2 Materials and methods

### 2.1 Participants and samples

We conducted a retrospective study. The tumor samples evaluated were obtained from a pool of patients diagnosed with PAs (n=117) at the neuroendocrinology outpatient clinic of the University Hospital of the Federal University of Maranhão – HUUFMA, São Luís, Maranhão, Brazil.

All patients included in the study were adults (≥ 18 years old) with a clinic diagnosis of PA, who had been submitted to transsphenoidal hypophysectomy, performed by the HUUFMA Neurosurgery team. During the follow-up, patients received drug treatment for tumor and/or biochemical control, according to the endocrinologist’s teams’ decision, before and/or after surgery: in the Acromegaly group, 18 patients received somatostatin analogs; in the Cushing’s disease group, 7 patients were treated with ketoconazole, and none of them received other drugs directed for hormonal secretion control; in the NF tumors, 34 patients with were treated with cabergoline for tumor growth control. Radiotherapy was indicated after surgery as advised by hospital guidelines. Patients under 18 years of age or whose histopathological analysis was incompatible with PA were not included in this study.

This research was approved by the ethics committee of the University Hospital of the Federal University of Maranhão, CAAE n° 95176418.5.0000.5086, according to the principles of the Declaration of Helsinki. Patients participated in the study after signing an informed consent form.

### 2.2 Clinical assessment

The diagnosis of PAs was established based on the clinical presentation, magnetic resonance imaging of the pituitary gland, and histopathological evaluation, in addition to a hormone panel evaluated by immunohistochemistry. Clinical and laboratory data were collected from medical records, and histopathological data were collected at the Pathological Anatomy Service at HUUFMA.

Patients were classified according to clinical data and following the most recent published guidelines, and 3 groups were included in the study: clinically non-functioning adenomas (NF), corticotropinomas (Cushing’s disease) and somatotropinomas (acromegaly) (29–31). Prolactinomas were not included in this study, since we had only 2 patients with prolactinoma submitted to neurosurgery, and one of them was under 18 years of age. There was no FSH-secreting tumor in our sample. The overall mean follow-up time was 72 months (5 patients were followed for < 1 year; 40 patients were followed for 1-5 years; 58 patients were followed for 5-10 years and 14 patients had a follow-up time > 10 years).

The variables used to characterize PAs aggressiveness were tumor size, number of surgeries, radiotherapy, need for medication to control the disease, regrowth after surgery, and biochemical control. For this last variable, the targets used were the normalization of serum IGF-1 in 2 measurements (acromegaly group) and suppression in the cortisol after dexamethasone-1 mg in at least 2 samples (Cushing’s disease group). Invasiveness was assessed by the modified (32). Cortisol and IGF-1 levels were evaluated by Chemiluminescent immunometric assay (Roche^®^). Six patients were submitted to radiotherapy: 3 in the NF tumor group, 2 in the Cushing group, and 1 in the Acromegaly group.

### 2.3 Nucleic acid extraction and cDNA synthesis

The isolation of total RNA was performed with the RNeasy Mini Kit (Qiagen) following the manufacturer’s recommendations. The nucleic acids obtained were quantified using a NanoDrop Lite spectrophotometer (Thermo Scientific). For cDNA synthesis, 1 μg of RNA was subjected to reverse transcription (RT−PCR) using a High Capacity cDNA Reverse Transcription Kit (Applied Biosystems) according to the manufacturer’s recommendations.

### 2.4 Analysis of the gene expression of *VDACs, BAX and BAK1*

The gene expression of *BAX, BAK1*, *VDAC1 and VDAC2* was evaluated by real-time quantitative PCR (qPCR). The RT-PCR reactions were carried out on a Rotor Gene Q Detection System (Quiagen) instrument using 100ng cDNA, TaqMan^®^ Universal PCR Master Mix (Applied Biosystems) and a predesigned and labeled primer/probe set (Assays-on-Demand™ Gene Expression Assay, Applied Biosystems), according to a previously described protocol (25). The following pre-designed TaqManH probes were used: *VDAC2* (Hs00748551_m1), *VDAC1* (Hs04978484_m1), *BAX* (Hs00180269_m1) and *BAK1* (Hs00940249_m1). All samples were analyzed in duplicate for each gene tested. Negative controls were included. Relative quantification of gene expression was calculated by the 2-DDCt method, the GAPDH was used as the reference gene. A pool of DNA extracted from 5 samples of normal pituitary tissue, with viability attested by an experienced pathologist, was used as the calibrator.

### 2.5 Statistical analysis

The data were processed using SPSS version 27.0 (IBM, Chicago, IL, USA) and GraphPad Prism version 9.5.1 (GraphPad Software, San Diego, CA, USA). Descriptive statistics included frequency, mean, median, standard deviation (± SD) and interquartile range (IIQ). The relative quantification (RQ) data of *VDAC1, VDAC2, BAX* and *BAK* gene expression were compared to the calibrator values (RQ = 1).

Chi-square or Fisher’s exact tests were applied to compare the frequency distributions of clinical and therapeutic data among the PA groups (acromegaly, Cushing’s disease and NF). The normality of the distribution of gene expression variables was assessed using the Shapiro–Wilk test. Due to the nonnormal distribution, nonparametric tests were selected. The Wilcoxon test (Wilcoxon signed-rank test) for an isolated sample was used to compare the RQ of the investigated category to the calibrator RQ. The Mann–Whitney test was used for the comparative analysis of gene expression in each category of clinical and therapeutic variables in each diagnostic group. Furthermore, the Spearman correlation coefficient (sr) was calculated to estimate the correlation strength between gene expression and PA diagnosis.

For all analyses, the significance level adopted was 5% (P <0.05).

## 3 Results

### 3.1 Demographic and clinical characterization

A total of 117 patients (48 men and 69 women) with a mean age of 48.5 years (± 13.2 years) were included in the study, with an average time since diagnosis of 2.5 ± 1.9 years (**Table 1**).

**Table 1:** Distribution of demographic and clinical variables in the sample of patients with pituitary adenoma.

Variables	mean	± SD	n	(%)
Sex				
Male			48	(41.0)
Female			69	(59.0)
Age (in years)	48.5	± 13.2		
Age group				
≤30 years			9	(7.7)
31 to 40 years			27	(23.1)
41 to 50 years			30	(25.6)
51 to 60 years			27	(23.1)
≥61 years			24	(20.5)
Clinical presentation of PA				
Acromegaly			24	(20.5)
Cushing’s disease			20	(17.1)
Nonfunctioning adenoma			73	(62.4)
Tumor size				
Micro			6	(5.1)
Macro			77	(65.8)
Giant			34	(29.1)

± SD, standard deviation; PA, pituitary adenoma.

The most common clinical presentation was NF adenoma (73 patients; 62.4%), followed by acromegaly (24 patients; 20.5%) and Cushing’s disease (20 patients; 17.1%). Most of the cases presented, at diagnosis, as macro (77/117-65.8%) or giant adenomas (34/117-29.1%) (**Table 1**).

With regard to the variables associated with the aggressiveness of PAs, 5.1% (6/117) of the patients who underwent radiotherapy and 21.4% (25/117) of the patients who underwent surgery had undergone more than one surgical procedure (**Table 2**).

**Table 2:** Distribution of variables related to the aggressiveness and invasiveness of pituitary adenomas.

Variable	n	(%)
Number of surgical procedures		
One	92	(78.6)
Two	19	(16.2)
Three	5	(4.3)
Four	1	(0.9)
Radiotherapy		
Yes	6	(5.1)
No	111	(94.9)
Use of medication for PA*^a^*		
Yes	25	(56.8)
No	19	(43.2)
Biochemical control*^a^*		
Yes	20	(45.5)
No	24	(54.5)
Tumor growth after surgery		
No enlargement	67	(57.3)
<10%	12	(10.3)
11 to 30%	10	(8.5)
31 to 50%	5	(4.3)
>50%	11	(9.4)
No data	12	(10.3)
Knosp classification		
0–2 (less invasive)	73	(62.4)
3–4 (more invasive)	44	(37.6)

PA, pituitary adenoma.

^a^Frequency calculated in the total sample of patients with Acromegaly or Cushing’s Disease.

Among the patients with acromegaly and Cushing’s disease, 56.8% (25/44) used medication to treat their PAs, and 45.5% (20/44) had achieved biochemical control.

Furthermore, 32.5% (38/117) of the patients experienced an increase in tumor size after surgery. The most invasive tumors (Knosp categories 3 and 4) represented 37.6% (44/117) of the sample (**Table 2**).

### 3.2 Distribution of clinical and therapeutic characteristics by diagnostic group

Concerning tumor size, our results showed significant differences between the groups (P <0.001) (**Table 3**). Microadenomas were mostly identified in the Cushing’s disease group (5/6–83.3%), while giant adenomas were predominant in the acromegaly and NF groups (33/34–97.0%).

**Table 3:** Distribution of the characteristics of aggressiveness and invasiveness according to the type of pituitary adenoma.

Variables	Acromegaly		Cushing’s disease		Nonfunctioning adenoma		*P*
	n	(%)	n	(%)	n	(%)	
Tumor size							<.001*^b^*
Micro	1	(4.2)	5	(25.0)	0	(0)	
Macro	18	(75.0)	14	(70.0)	45	(61.6)	
Giant	5	(20.8)	1	(5.0)	28	(38.4)	
Number of surgical procedures							.768
One	19	(79.2)	17	(85.0)	56	(76.7)	
Two or more	5	(20.8)	3	(15.0)	17	(23.3)	
Radiotherapy							.416
Yes	1	(4.2)	2	(10.0)	3	(4.1)	
No	23	(95.8)	18	(90.0)	70	(95.9)	
Use of medication for PA*^a^*							.007*^b^*
Yes	18	(75.0)	7	(35.0)	–	–	
No	6	(25.0)	13	(65.0)	–	–	
Biochemical control*^a^*							.245
Yes	9	(37.5)	11	(55.0)	–	–	
No	15	(62.5)	9	(45.0)	–	–	
Tumor growth after surgery							.519
No	17	(73.9)	12	(60.0)	38	(61.3)	
Yes	6	(26.1)	8	(40.0)	24	(38.7)	
Knosp classification							.092
0–2	18	(75.0)	15	(75.0)	40	(54.8)	
3–4	6	(25.0)	5	(25.0)	33	(45.2)	

PA, pituitary adenoma.

^a^Frequency calculated in the total sample of patients with Acromegaly or Cushing’s Disease.

^b^P less than.05.

Considering the use of medication, a greater rate of achieving biochemical control was observed in acromegaly patients than in patients with Cushing’s disease (P = 0.007) (**Table 3**).

### 3.3 Relative gene expression of the *VDAC1, VDAC2, BAX*, and *BAK1* according to the clinical presentation of PA

**Figure 1:**
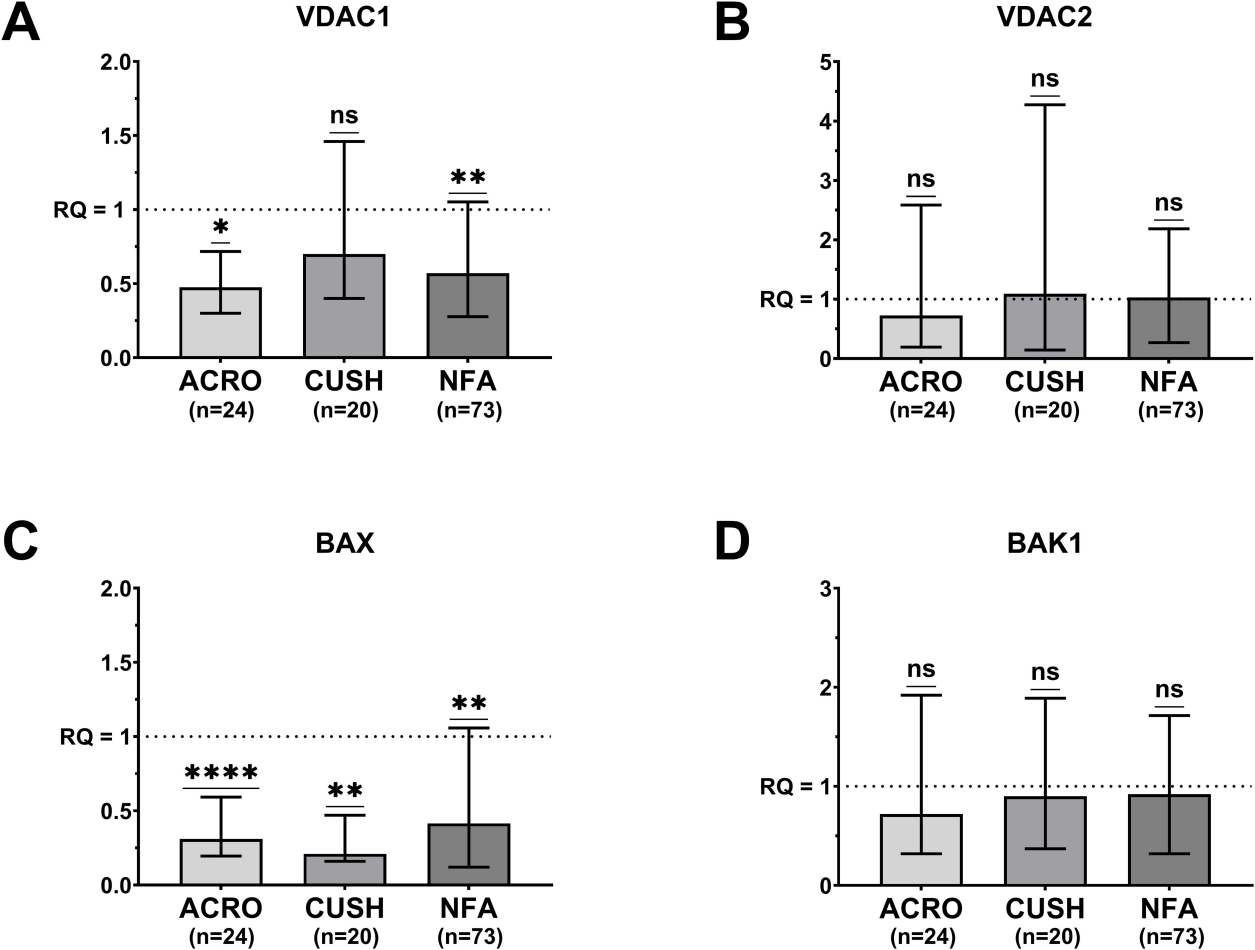
Median (bar) and interquartile range (whiskers) of differential gene expression of *VDAC1* **(A)**. 660 *VDAC2* **(B)**. *BAX* **(C)** and *BAK1* **(D)** according to pituitary adenoma type. 661 ACRO, acromegaly group; CUSH, Cushing’s disease group; NFU, non-functioning 662 adenoma group; One sample Wilcoxon test; ns, not significant; *P <.05. **P <.01. ****P 663 <.0001.

A significant down-regulation of *VDAC1* gene expression was observed in the acromegaly (median RQ = 0.47; P = 0.029) and NF groups (median RQ = 0.57; P = 0.002) compared to the normal pituitary tissue (**Figure 1A**). *BAX* expression was significantly lower in all patients: acromegaly (median RQ = 0.31; P <0.001), Cushing’s disease (median RQ = 0.21; P = 0.007) and NF (median RQ = 0.41; P =0.005) (**Figure 1C**).

The expression of the *VDAC2* and *BAK1* genes in the three PA groups did not differ from that in the normal control (**Figures 1B, D**).

### 3.4 Correlations between the relative expressions of the *VDAC1, VDAC2, BAX, and BAK1* genes by group

**Figure 2:**
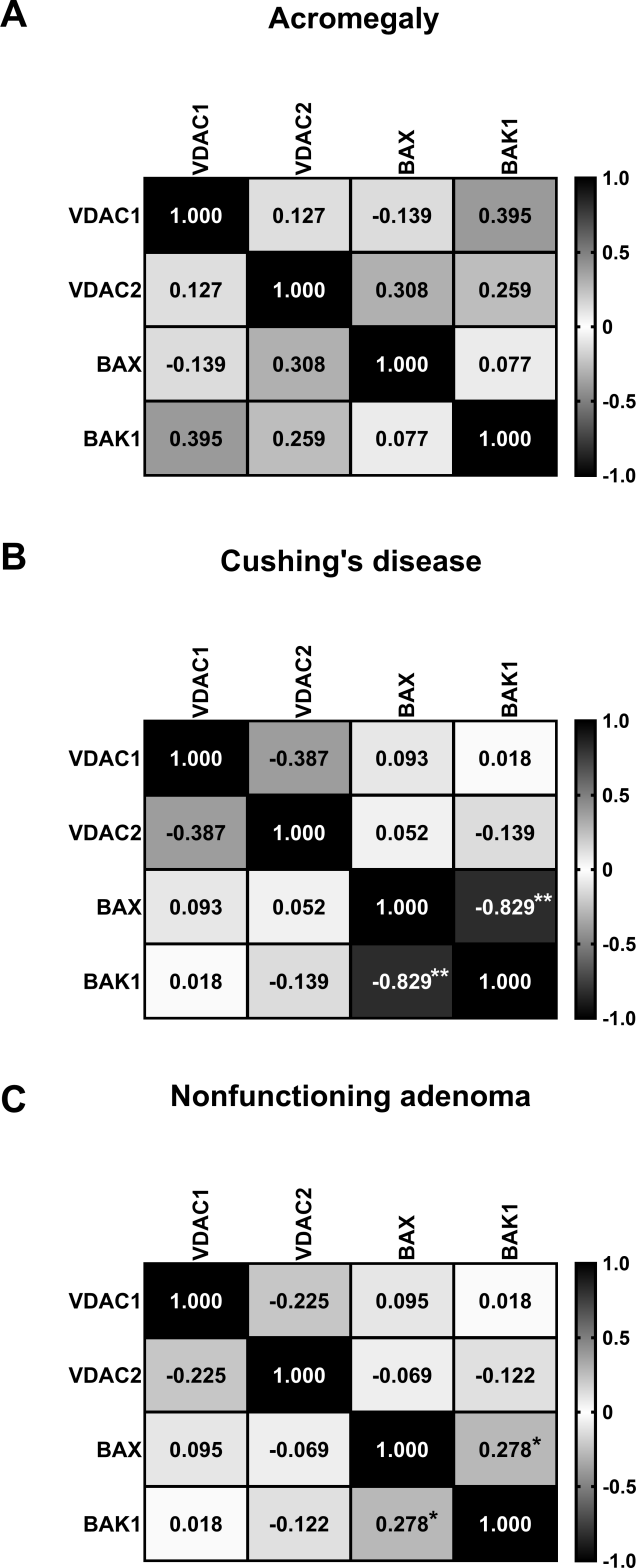
Matrix showing Spearman’s correlation coefficients between the relative quantifications of VDAC1 and VDAC2 gene expression in acromegaly group **(A)**, Cushing’s disease group **(B)**, and nonfunctioning adenoma group **(C)**. *P <.05. **P <.017777.

In the acromegaly group, no significant correlations were detected between gene expression levels (**Figure 2A**). In the Cushing’s disease group, a strong inversely proportional correlation was observed between *BAX* and *BAK1* expression levels (rs = -0.829; P = 0.005) (**Figure 2B**). In the NF group, a weak direct correlation was identified between the expression levels of *BAX* and *BAK1* (rs = 0.278; P = 0.048) (**Figure 2C**).

### 3.5 Differential relative gene expression of VDAC*1, VDAC2, BAX*, and *BAK1 according* to the clinical behavior of the tumor

The analysis of gene expression based on tumor size showed that in macroadenomas, both *VDAC1* and *BAX* were decreased (median RQ = 0.49; p <0.001 and median RQ = 0.43; p <0.001, respectively). In giant tumors, only *BAX* showed decreased expression levels (median RQ = 0.18; p <0.014). However, microadenomas did not exhibit changes in any of the analyzed genes compared to the control tissue (**Table 4**).

**Table 4:** Differential expression of VDAC1, VDAC2, BAX and BAK genes according to pituitary adenoma size and invasiveness.

Variables	*VDAC1*		*VDAC2*		*BAX*		*BAK1*	
	RQ med	*P*	RQ med	*P*	RQ med	*P*	RQ Med	*P*
Tumor size								
Micro	1.60	.437	1.51	.437	0.32	.625	0.80	.875
Macro	0.49	<.001*^a^*	1.03	.149	0.43	<.001*^a^*	1.03	.257
Giant	0.74	.467	0.73	.835	0.18	.014*^a^*	0.53	.465
Tumor growth after surgery								
Yes	0.58	.097	1.46	.042	0.34	.054	0.64	.724
No	0.57	.001*^a^*	0.69	.855	0.32	<.001*^a^*	0.92	.561
Knosp classification								
0–2	0.57	<.001*^a^*	0.82	.628	0.32	<.001*^a^*	0.89	.713
3–4	0.59	.343	1.39	.066	0.36	.005*^a^*	0.83	.707

RQ med, median of the relative quantification of gene expression compared to control (RQ = 1).

^a^Indicates statistically significant differences compared to the control (P less than.05). P value calculated using the one sample Wilcoxon test.

In relation to tumor growth after surgery, on the contrary, a significant increase in *VDAC2* expression was observed in patients who experienced postsurgical regrowth (median RQ = 1.46; p = 0.042), while the response of the other genes showed a trend towards downregulation, although without statistical significance (**Table 4**).

Furthermore, the downregulation of *BAX* expression was noted regardless of the degree of invasiveness measured by the Knosp classification (median RQ = 0.32; p<0.001). However, only *VDAC1* presented a down-regulation (median RQ = 0.57; p<0.001) in the minor degree of this classification (0-2) (**Table 4**).

*BAK1 gene* expression was not related to any category of clinical manifestation or biological behavior of PAs included in this study.

## 4 Discussion

PAs are benign tumors with a high frequency in the population and occasionally aggressive behavior (8). The poor understanding of the biopathology of these tumors has hindered the establishment of efficient prognostic markers, limiting their clinical management. Studies have demonstrated the participation of genes related to cell replication and apoptosis in the pathogenesis of several malignant neoplasms; however, in PAs, this process is still unknown (33, 34). To address this topic and identify potential new markers, this work evaluated, for the first time, the combined gene expression of *VDAC1, VDAC2, BAX* and *BAK1*, which are fundamental components of apoptosis, in normal pituitary tissue and in different subtypes of PAs.

### 4.1 Clinical analysis

The sample was mainly composed of women, with a mean age of 48 years, and macroadenomas were relatively more common, which is similar to the findings of other studies (35, 36). Regarding the clinical type, almost two-thirds of the sample consisted of NF adenomas (62.4%). These findings are consistent with other studies showing that this clinical type is 2 to 3 times more prevalent than somatotrophic and corticotrophic tumors, especially among giant tumors, which often present with compressive symptoms and are difficult to resect (1, 37). The frequency of giant tumors in our study (29.1%) was greater than that previously reported (6-10%) (35, 38, 39), which can be explained, in part, by the difficulty in accessing diagnosis and treatment promptly in our country.

In the present study, 45.2% of the NF adenomas were invasive at diagnosis, compared to 25% in the Cushing’s disease and acromegaly groups. Other studies reported frequencies of Knosp 3-4 tumors of 10.8-12% in Cushing’s disease patients (40, 41) 14.7-29% in acromegaly patients (42, 43) and 26.5-29% in NF adenoma patients (44, 45).

Acromegaly was the second most prevalent subtype in our sample (20.5%), with the majority (95.8%) of these tumors classified as macro- or giant tumors at diagnosis. Other studies have reported a proportion of macroadenomas at presentation ranging from 66 to 76.5% (43, 46). In our study, there was also a greater need for medication use to control the disease since 75% of the participants used somatostatin analogs (SAs) and/or cabergoline. Biochemical control was achieved in 37.5% of the patients, which could explain the resistance profile of these tumors. Our result is in agreement with those of other studies, which showed normalization of IGF-1 in 23 to 40% of patients using SA (47, 48). In the group using combination therapy (cabergoline + SA), biochemical control was demonstrated in 37-56% of the patients (49, 50).

The Cushing’s disease group had a greater frequency of microadenomas than did the other groups; nevertheless, the Cushing’s disease group had a regrowth rate comparable to those of the acromegaly and NF adenoma groups, whose rates of giant tumors were greater. Additionally, in the Cushing’s disease group, 25% of the tumors were invasive (Knosp 3-4). As previously reported, corticotroph adenomas often present as small tumors, with recurrence rates ranging from 28 to 32%, which is similar to our findings (51, 52).

In this context, the presence of regrowth after surgery represents an important clinical marker of PAs aggressiveness (53). In our sample, 32.4% of tumors regrew, with similar rates among the different groups. Several studies have demonstrated regrowth rates ranging from 20 to 50%, notably in patients with mutations associated with aggressive corticotroph tumors (44, 54–56). It is important to identify markers that can predict tumor regrowth after surgery to improve the treatment of patients with PAs.

### 4.2 *VDAC1* and *VDAC2* analysis

In this study, we found lower *VDAC1* expression in acromegaly and NF adenomas than in normal pituitary tissue. These clinical groups had the largest proportions of giant tumors in our sample; therefore, this finding may suggest that the decreased expression of *VDAC1* in these tumors may induce resistance to apoptosis, resulting in larger tumors, as previously suggested for other proapoptotic genes (57, 58).

The expression of VDACs in benign tumors is not yet well established. To our knowledge, the studies of *VDAC*’s actions in apoptosis are restricted to malignant tumors, in which the results are divergent. As an example, in Cholangiocellular Carcinomas, Feichtinger et al. (58) showed underexpression of *VDAC1* in tumors with aggressive behavior and suggested that the high dependance on glucose of these aggressive tumors could be responsible for reduced mitochondrial mass, and lower *VDAC1* expression. In malignant tumors, otherwise, overexpression of *VDAC1* was observed, notably in those with a more severe clinical course, which, according to some authors, could be associated with adaptive changes in the energy metabolism of cancer cells, with increased aerobic glycolysis (Warburg effect) that results in greater resistance to cell death (19, 22, 27, 59–63). Mazure et al. (16) highlighted the importance of the interaction of *VDAC* and the *Hexokinases*, which are more expressed in several types of cancer, and have an important role in the acquisition of energetic advantages, magnifying glycolysis and mitochondrial metabolism.

These differences between *VDAC1* expression in cancers (overexpression) and in PAs (underexpression) may occur due to the distinct effects of this gene on apoptosis in these tumors; however, further studies on *VDAC1* expression in pituitary carcinomas may contribute to a greater understanding of these findings.

There was no significant difference in *VDAC2* expression compared to that in normal pituitary tissue among the 3 subgroups of PAs. There have been no studies on *VDAC2* expression in benign PAs. The results reported in the literature regarding *VDAC2* expression in malignant neoplasms are inconsistent (65). *VDAC2* overexpression has been detected in 2 subtypes of differentiated thyroid carcinomas, and its silencing led to a better clinical response to chemotherapy (25). In contrast, another study demonstrated that the deletion of *VDAC2* in rodent glioblastomas and colorectal tumors inhibited *BAX-*mediated apoptosis and worsened the response to chemotherapy, suggesting that *VDAC2* may be essential for activating BAX-mediated apoptosis and limiting the growth of these tumors (24). Unlike the findings in malignant tumors (24, 27, 64), our results did not show a correlation between *VDAC2* and *BAX* or between VDAC2 and *BAK1* in PAs. Nonetheless, interestingly, *VDAC2* was overexpressed in tumors that regrew after surgery, regardless of the tumor subgroup. This observation suggests a potential role for *VDAC2* as a prognostic marker of aggressive behavior. It is possible that Increased expression of *VDAC2* induces proliferative behavior and reduces apoptosis in PAs, as observed in other neoplasms (23, 64). Other studies have suggested that the ability of *VDAC2* to induce apoptosis and control tumor growth appears to depend on its interaction with *BAK* and *BAX* (23–25); however, our results did not show any correlation in the expression of these genes.

### 4.3 *BCL-2* family analysis

For *BAX*, underexpression was evident in all PA subtypes evaluated. Therefore, it is assumed that resistance to apoptosis mediated by *BAX* may be one of the mechanisms involved in pituitary tumorigenesis since this gene acts as an effector of apoptosis in several neoplasms (61, 65–67). These findings are in concordance with the results showed by Ozer et al., which evaluated the immunohistochemical expression of BAX in a group composed mainly of macroadenomas and verified the decreased expression of BAX in recurrent tumors, indicating that this protein plays an important role in preventing apoptosis in PAs (68).

There were no significant differences in the expression of *BAK1* in the different subtypes of PA. Like *BAX*, *BAK1* has a proapoptotic role, and an increase in its expression in lung tumors is associated with decreased resistance to therapy (69); however, our results suggest that this gene could be less important for pituitary tumorigenesis.

A strong negative correlation was also detected between *BAX and BAK1* expression in corticotrophic tumors, and a weak positive correlation was detected in NF adenomas. *BAK1* and *BAX* are structurally and functionally similar, and their expression is influenced by several factors (70). It is possible that the role of these genes in PAs is determined by interrelationships with other genes (66, 70, 71) not studied in this paper. Furthermore, we did not identify any other studies that have specifically evaluated the interaction between *BAK1* and *BAX* in PAs, and differences in these relationships according to clinical subtype are not yet understood.

### 4.4 Invasiveness analysis

Regarding invasiveness, when comparing invasive (Knosp 3-4) and noninvasive (Knosp 0-2) tumors, no difference was found in the expression of the genes studied. This finding may suggest a limited degree of influence of these genes on the invasiveness of PAs, which may be linked to the existence of pathways not directly related to apoptosis mediated by the investigated genes (72).

### 4.5 Limitations

The main limitations of this work are related to the difficulty in accessing treatment for patients in our country, which may have contributed to the small proportion of patients with microadenomas in the sample, making comparisons between groups difficult. It is also worth noting the limited number of patients undergoing radiotherapy due to the technical limitations of this therapeutic modality in our region, which made it difficult to assess the impact of the studied markers on the response to this type of treatment.

### 4.6 Conclusion

In conclusion, the *VDAC1, VDAC2*, and *BAX* genes appear to be involved in the biopathology of PAs, and their expression levels may help to define subgroups with different risks of postsurgical regrowth. In particular, the decreased expression of *VDAC1* and *BAX* may suggest a role for these genes in resistance to apoptosis in PAs. The expression of *VDAC2* was shown to increase in PAs with regrowth, which may suggest, as previously reported in other neoplasms, an antiapoptotic action of *VDAC2* in PAs. Functional studies in experimental models and future clinical studies with larger sample sizes are necessary to understand the role of *VDACs, BAK1* and *BAX* in pituitary tumorigenesis and their potential aggressive behavior, in addition to other markers associated with this process, giving rise to new therapeutic targets for PAs.

## Data Availability

The raw data supporting the conclusions of this article will be made available by the authors, without undue reservation.
